# Supplementary material for: Parameters estimation of stochastic finite fault ground motion simulation method and its application in North China
Source: PLoS One. 2025 Dec 3;20(12):e0337602. doi: 10.1371/journal.pone.0337602 (PMC12674566; doi:10.1371/journal.pone.0337602)
Supplement: S2 File — The program code of strong ground motion simulation based on stochastic finite-fault method with dynamic corner frequency for the Pingyuan earthquake. (ZIP) [file pone.0337602.s002.zip › S2 File. Program Code. ( Simulationor of Pingyuan earthquake)/EXSIMNC/EXSIM12-readme.pdf]

EXSIM12: A stochastic finite-fault computer program in FORTRAN

Karen Assatourians and Gail Atkinson, May 2012

Modified from the EXSIM code of Motazedian and Atkinson (2005), incorporating the improvements suggested by Boore (2009). Original FINSIM code by Beresnev and Atkinson (1998). The program is run from a DOS window (within the directory containing the program and its input files) by typing EXSIM12

The files in this directory include the FORTRAN source (EXSIM12.for) and executeable (EXSIM12.exe) codes for EXSIM12. All needed subroutines are directly embedded in the source code. EXSIM12 is a stochastic finite-fault algorithm to generate acceleration time series for specified earthquake fault rupture scenarios, where the ruptures are specified by a few simple metrics such as earthquake magnitude and distance, with options to include more detailed information on fault geometry and slip, or net propagation effects, as available. See the references for the methodology. Comments on program operations are contained within the source code. The main loops for the program operations are shown below.

Required input files:

Input parameter file (main input file): see the example file **Example_EXSIM12.par**

Site amplification file (specifies site amplifications to be applied): see the example file **site_amps.txt**

Crustal amplification file (specifies crustal amplification to the base of the site soil profile): see the example file **crustal_amps.txt**

Empirical amplification file (allows multiplication by an empirical filter to fine-tune the specified spectrum as per Atkinson et al., 2011): see the example file **empirical_amps.txt**

Note that the Fourier spectrum that generates the time series includes the product of the site, crustal and empirical amplifications.

Generated output files:

Acceleration time series: **ACC.out** gives the 1st simulated trial for each case in a single output file in the root directory. The subdirectory **ACC** contains all of the generated trials.

Response and Fourier Spectra: the subdirectory **PSA** contains average output spectra (over all trials for a given scenario) in the *.out files. Values for individual trials are given in the *.tmp files

Other: The subdirectory **other** provides other information including modeling parameters, Husid parameters, Arias intensity and duration parameters.

References

Atkinson, G., K. Goda and K. Assatourians (2011). Comparison of nonlinear structural responses for accelerograms simulated from the stochastic finite-fault approach versus the hybrid broadband approach. Bull. Seism. Soc. Am., **101**, 2967-2980

Beresnev, I. and G. Atkinson (1998). FINSIM - a FORTRAN program for simulating stochastic acceleration time histories from finite faults. Seism. Res. L., **69**, 27-32.

Boore, D. (2009). Comparing stochastic point-source and finite-source ground-motion simulations: SMSIM and EXSIM. Bull. Seism. Soc. Am., **99**, 3202-3216.

Motazedian, D. and G. Atkinson (2005). Stochastic finite fault modeling based on a dynamic corner frequency, Bull. Seism. Soc. Am., **95**, 995-1010

Main EXSIM12 Program Loops

Line	Loop	Description
322:	do while (.not. f_exist)	Getting input file name
340:	end do	
419:	do k=2,nfreq	Generate logarithmically spaced frequencies
421:	enddo	
448:	do i = 1, nfout	Make frequency part of headlines in summary PSA files
454:	end do	
472:	DO isite=1,numberOfSites	
513:	DO ihypo = 1, n_hypocenters	
531:	DO i=1,nl	Calculate some parameters of sub-sources: f_0, slip, moment, duration, …
532:	DO j=1,nw	
595:	END DO	
596:	END DO	
641:	DO isim=1,nsims	
650:	DO i=1,nl	Time series generated and summed
651:	DO j=1,nw	
692:	DO k = 1, nhigh	Add current sub-source accelerogram to the total
694:	END DO	
698:	END DO	
699:	END DO	
733:	do i = 1, npw2TotalWave	Husid calculation
735:	end do	
823:	END DO	Individual simulations: *.acc files + *husid* files + *.tmp files (spectra)
831:	DO i = 1, nfreq	Average spectra calculation
874:	END DO	
903:	END DO	Summary PSA / duration / arias intensity files (*dur*dist_psa.out)
911:	DO i = 1, nfreq	Average PSA/FAS
916:	END DO	
919:	do i = 1, nfreq4intrp	Interpolation for average PSA/FAS
922:	end do	
924:	do i = 1, nfout	
935:	end do	PSA/FAS files written here (*psa_fs_*.out)
963:	END DO	Summary PSA / duration / arias intensity files (*dur*dist_psa.out)

Individual simulations:

- *.acc files +
- *husid* files +
- *.tmp files (spectra)

PSA/FAS files written here (*psa_fs_*.out)

Summary PSA / duration / arias intensity files (*dur*dist_psa.out)
