# Supplementary material for: Parameters estimation of stochastic finite fault ground motion simulation method and its application in North China
Source: PLoS One. 2025 Dec 3;20(12):e0337602. doi: 10.1371/journal.pone.0337602 (PMC12674566; doi:10.1371/journal.pone.0337602)
Supplement: Attachment — Submitted filename: Response to reviewers---1004.pdf [file pone.0337602.s005.pdf]

PLOS ONE

Ref: Submission ID PONE-D-25-27507

Title: Parameters estimation of stochastic finite fault ground motion simulation method and its application in North China

Response: Letter to Reviewer' Comments

Comments from the Editor:

We thank all the editors and reviewers for their valuable comments and suggestions. We have carefully revised the manuscript to enhance its clarify and facilitate the understanding of the readers. Our point-to-point responses are presented in the following. We hope that the revision would satisfactory address the comments and concerns of the editors and reviewers.

1. Thank you for your response to our Data Availability Statement.

We have rewritten your Data Availability Statement as follows based on the provided information. Please can you read and approve of the below statement. We will then be able to update your Data Availability Statement and update it accordingly.

"Yes - all data are fully available without restriction;

The finite-fault ground motion simulation program code can be downloaded from website of Engineering Seismology Toolbox (https://www.seismotoolbox.ca/EXSIM12.html). Some seismic parameters are available at website of USGS (https://earthquake.usgs.gov/earthquakes/eventpage/us6000ky5l/executive). Strong motion recordings for this study are provided by China Strong Motion Network Centre at Institute of Engineering Mechanics, China Earthquake Administration."

Response: Yes, I approve of the Data Availability Statement and update in my manuscript.
