# Supplementary material for: Parameters estimation of stochastic finite fault ground motion simulation method and its application in North China
Source: PLoS One. 2025 Dec 3;20(12):e0337602. doi: 10.1371/journal.pone.0337602 (PMC12674566; doi:10.1371/journal.pone.0337602)
Supplement: Attachment — Submitted filename: Comments.docx [file pone.0337602.s003.docx]

The authors estimated some input parameters, such as site amplifications, high-frequency attenuation and stress drop, in the study area, and simulated the PGA, FAS, acceleration time history, PSA, and shake map of PGA for the Dezhou earthquake. The simulated results coincided well with observed values. However, some issues should be addressed before the manuscript can be published.

1.Lines 39 and 71, peak ground acceleration (PGA)?

2.Line 97, GB50011-2010^15^ ?

3.Equation (2) is not correct. (1+(*f*/*f_oij_*)^2^).

4.Parameter *H_ij_* in equation (2) is not defined.

5.Line 155. The authors claimed that some modifications or variation of EXSIM12 on some input model parameters. These are only estimation of regional parameters based on seismic records in the study region, and are not improvements to the EXSIM model. The author should revise the relevant descriptions throughout the entire text.

6.Line 181, region^21^, ?

7.Equation (6) is not correct, kappa is defined as the slope, which is calculated by the least square method, instead of directly calculating the slope using κ = -(lnA(f2)-lnA(f1))/( π(f2-f1)).

8.Line 229, κ = k/(πloge) may be wrong. If the FAS curve was plotted in a semi-logarithmic space, the parameter κ can be obtained by the following equation κ= k/π. Equation (6) contradicts the equation in line 229.

9.Lines 228 and 230, Kappa	kappa.

10.Line 295, Motazedian^14^, ?

11.Line 298, Atkinson and Boore^1^, ?

12.Figure 3(c) and (d), what is the reason for a negative slope?

13. Line 53, Boore^13^, ?
