# Supplementary material for: Parameters estimation of stochastic finite fault ground motion simulation method and its application in North China
Source: PLoS One. 2025 Dec 3;20(12):e0337602. doi: 10.1371/journal.pone.0337602 (PMC12674566; doi:10.1371/journal.pone.0337602)
Supplement: Attachment — Submitted filename: Response to reviewers-1102.docx [file pone.0337602.s006.docx]

**PLOS ONE**

**Ref**: Submission ID PONE-D-25-27507

**Title**: Parameters estimation of stochastic finite fault ground motion simulation method and its application in North China

**Response: Letter to Reviewer’ Comments**

**Comments from the Editor:**

We thank all the editors and reviewers for their valuable comments and suggestions. We have carefully revised the manuscript to enhance its clarify and facilitate the understanding of the readers. Our point-to-point responses are presented in the following. The changes in the revised manuscript have been highlighted. We hope that the revision would satisfactory address the comments and concerns of the editors and reviewers.

1. Both reviewers acknowledge the effort made in revising the manuscript, but they still identify important issues that need to be addressed before the paper can be reconsidered for publication.

**Response:** Thanks for your positive comments.

As can be seen in the Revised Manuscript with Track Changes, we have carefully revised this manuscript based on the important issues and other issues reviewers identified. We hope that the revisions and improvements would address the reviewers’ concerns satisfactorily.

2. The reviewers emphasize that the study does not introduce fundamental improvements to the stochastic finite-fault method, but rather focuses on the regional calibration of parameters such as site amplification, kappa, and stress drop. Therefore, the title and text should accurately reflect the nature of this contribution, avoiding the use of expressions such as “improved method” or “improved model.”

**Response:** We fully agree with the reviewers’ assessment. As the reviewers mentioned, this paper focuses on some region-specific key parameters including local site amplification, high-frequency decay factor kappa and stress drop. In fact, we have done little work on the essential and fundamental improvements to the stochastic finite-fault method.

According to the reviewers’ advice, the expressions such as “improved method” or “improved model” have been deleted or changed in full paper. On the basis of reading and revising the entire manuscript, the expressions such as “improved method” or “improved model” have been revised.

Please refer to the revised manuscript for details on multiple revisions.

3. Additional clarification is required regarding the observed linear increase of the amplification coefficient with frequency in logarithmic coordinates, as well as the methodology used for the estimation of kappa. The equations presented in the manuscript appear inconsistent, and it should be clearly stated which formulation was used, ensuring that the correct expression is applied throughout. The differences observed in the fitted lines of Figure 3 also need to be explained.

**Response:** Thanks for the valuable comment.

The observed linear increase of the amplification coefficient with frequency in logarithmic coordinates has clarified in the following text. In logarithmic coordinates, the line is plot form the ten points. The phenomenon of linear increase with frequency are attributed to the limited number of point observations. The linear increase line segment is the line connecting from two adjacent points.

In the methodology used for the estimation of kappa, we used the equation (6), rather than equation defined in line 238. We have deleted the sentence in line 238.

The differences observed in the fitted lines of Figure 3 has been explained and carefully clarified in the in section “**Reviewer # 2”**.

By checking our drawing data and drawing programs in Matlab, we find that the linear fitted lines and negative slope in original Figure 3(c) and (d) are false. The figure 3 we presented is the original research picture.

We are so sorry that I forget to provide new picture in the manuscript.

The data of κ value and program are checked and verified. Based on more κ value from horizontal components of all recordings, we re-fitted the data points linearly and obtained a linear form in research, but we did not provide these.

The new figure 3 we plot are given as the following, and we provide these in the second round revision.

In the new figure 3 ,the kappa value are re-calculated and the line are re-plot. The new linear fitted lines are given.

4. Reviewer 2 notes that some comments from the first review round (specifically comments 8, 9, and 12) remain insufficiently addressed. Please review these points carefully and provide a detailed response. It is also recommended that you discuss your results in the context of recent studies that have advanced the EXSIM methodology, explaining how your regional calibration complements rather than replaces those developments.

**Response:** We were really so sorry for our careless mistakes in the first review round. As the reviewer’ mentioned, the comments 8, 9 and 12 were addressed incompletely and insufficiently. For these comments, detailed responses and modifications have been made in the following text, as can be seen in section “**Reviewer # 2”.**

Compare to the literature that have advanced the EXSIM methodology, our work mainly concentrated on the region-specific key parameters in North China. In deed, we acknowledged that we have did little work on the development or improvement of EXSIM methodology. In fact, this important issue perfectly coincides with the assessment 2 from reviewer 1. Regarding this issue, we have explained our regional parameters calibration rather than method developments based on reading the entire text thoroughly. Please refer to the revised manuscript for details on multiple revisions.

5. To ensure your figures meet our technical requirements, please review our figure guidelines: https://journals.plos.org/plosone/s/figures

You may also use PLOS’s free figure tool, NAAS, to help you prepare publication quality figures: https://journals.plos.org/plosone/s/figures#loc-tools-for-figure-preparation.

NAAS will assess whether your figures meet our technical requirements by comparing each figure against our figure specifications.

**Response:** In this review round, in order to make the figures can meet the journal technical requirements, we have read the figure guidelines from the website carefully.

And, to ensure our figures in the paper meet publication quality, figure tool NAAS was adopted to process the original figures.

Finally, the original images and compliant figures in the paper are provided simultaneously.

In addition, the original images were created in a PDF file named ‘S1_raw_images’ as supporting information files.

**Reviewer # 1:**

1. Line 272: This study does not introduce fundamental improvements to the stochastic finite-fault method. Instead, it calibrates regional parameters such as local site amplification, high-frequency attenuation factor kappa, and stress drop based on borehole data and records from strong-motion stations. The calibrated parameters are then used for simulations, thereby improving the simulation accuracy. Therefore, we strongly suggest that the authors reconsider whether the current title accurately reflects the work.

**Response:** Thanks for your positive comments. We have carefully revised this manuscript based on the reviewer’s comments.

We fully agree with the reviewers’ assessment. As the reviewers mentioned, this paper does not essentially introduce fundamental improvements in method itself. In fact, we focus on some region-specific key parameters including local site amplification, high-frequency decay factor kappa and stress drop in North China. In addition, the parameters are used to simulate and improve the simulation accuracy. We have reconsidered the title and some expressions to actual reflect our work.

According to the reviewers’ advice, the expressions such as “improved method” or “improved model” have been deleted or changed in full paper. On the basis of reading and revising the entire manuscript, the expressions such as “improved method” or “improved model” have been revised.

Please refer to the revised manuscript for details on multiple revisions.

The title and sentence in Line 272 have been modified as follows:

Validation of the region-specific parameters

In this study, we make some comparisons between the finite-fault modeling in North China (EXSIM_NC) and previous simulation method (EXSIM_12). The Dezhou earthquake is taken as an example for the validation of the region-specific key parameters obtained in this paper.

2. Lines 187–189: Why does the site amplification coefficient obtained in this study exhibit a linear increase with frequency in logarithmic coordinates? Please clarify the underlying mechanism responsible for this phenomenon

**Response:** Thanks for the valuable comment.

In our study, the local amplification function over versus frequency for class II and III site are computed using the quarter-wavelength approximation.

For a particular frequency, the amplification value is calculated by the square root of ratio between the seismic impedance of bedrock at the depth of source and the average seismic impedance from surface to a quarter wavelength, where seismic impedance is defined as shear wave velocity times density. The algorithm expression is the following:

$\text{A(f)=}\sqrt{{\text{ρ}_{\text{s}}\text{β}_{\text{s}}}/{{\bar{\text{ρ}}}_{\text{z}}{\bar{\text{β}}}_{\text{z}}}}$	(4)

where $\text{ρ}_{\text{s}}$, $\text{β}_{\text{s}}$ represent density and shear wave velocity at source, and ${\bar{\text{ρ}}}_{\text{z}}$, ${\bar{\text{β}}}_{\text{z}}$ represent average density and shear wave velocity at site, respectively.

The quarter-wavelength approximation early used in the following paper, and we refer to this literature in our study.

[24] Boore DM, Joyner WB. Site amplifications for generic rock sites. Bulletin of the Seismological Society of America. 1997; 87(2): 327–341.

As computed and described in the literature [24], the amplification value versus frequency in the literature [24] were presented in the following picture.

In our study, based on the borehole shear wave velocity profile versus depth in North China, we calculated the the amplification value versus frequency. Our research results in North China are as following and can be seen in the Supporting Information.

In figure 3, we plot amplification coefficient obtained in this study with frequency by using the data in the red rectangular box. For example, this is the mean amplification function values estimated in North China.

In logarithmic coordinates, the line is plot from the ten points. The phenomenon of linear increase with frequency are attributed to the limited number of point observations. The linear increase line segment is the line connecting between two adjacent points.

We hope that the clarification for this phenomenon would address the reviewers’ concerns satisfactorily.

**Reviewer # 2:**

The authors have addressed most of the comments. However, the manuscript still has serious issues that need to be clarified by the authors.

**Response:** Thanks for the positive comment. We appreciate your summary of the manuscript and encouraging comment. Thanks for your positive comments. We have carefully revised this manuscript based on the reviewer’s comments. We hope that the revisions and improvements would satisfactory address the reviewers’ concerns.

1. The comments 8, 9 and 12 suggested in the first round is not addressed completely.

**Response:** We were really so sorry for our careless mistakes in the first review round. As the reviewer’ mentioned, the comments 8, 9 and 12 were addressed incompletely and insufficiently. We checked comments in first round and revised carefully as following.

First round 8: 8.Line 229, κ = k/(πloge) may be wrong. If the FAS curve was plotted in a semi-logarithmic space, the parameter κ can be obtained by the following equation κ= k/π. Equation (6) contradicts the equation in line 229.
**Response:** Thanks for your valuable comments.

In the kappa estimation, we used the equation (6), rather than equation defined in line 229 in first round.

The equation defined in line 229 ( in the first round ) comes from the literature:

(3) An updated stochastic finite fault modeling: Application to the Mw 6.0 earthquake in Jiashi, China. Soil Dynamics and Earthquake Engineering, 2022, 162:107450..

In our paper, the expression of the sentence is referred.

In revised manuscript, the sentence has been deleted as the following:

~~In Fourier amplitude spectrum diagram, the~~ $\text{κ}$ ~~of one ground motion recording is computed by~~ $\text{κ}$~~=k/(~~$\pi$~~loge), where k is the slope and e represents natural constant.~~

As mentioned by reviewer, we use Equation (6) in the semi-logarithmic coordinate system .

First round 9: 9.Lines 228 and 230, Kappa should be kappa.

**Response:** Thanks for your valuable comments.

The Kappa have been verified into kappa (*κ*) in the full paper.

Especially in the section 4.2 kappa (*κ*), we modified the false Kappa, and verified into *κ .*

As can be seen in the Revised Manuscript, the section has been modified as follows:

First round 12: 12.Figure 3(c) and (d), what is the reason for a negative slope?

**Response:** Thanks for your valuable and insightful comments.

By checking our drawing data and drawing programs in Matlab, we find that the linear fitted lines and negative slope in original Figure 3(c) and (d) are false .

We are so sorry that I forget to provide new picture in the manuscript. I think that the negative slope in original picture is false.

New data of κ value and program are collected and verified. Based on more κ data from horizontal components of all recordings, we re-fitted the data points linearly and obtained a new linear form.

The new figure 3(c) and (d) we plot are given as the following, which shows the positive slope.

We hope the revisions would satisfactory address the reviewers’ concerns.

2. In the Figure 3, the kappa values calculated from records are the same, but the linear fitted lines are different, why?

**Response:** Thank you for pointing this out.

By checking our drawing data and drawing programs in Matlab, we find that the linear fitted lines and negative slope in original Figure 3(c) and (d) are false. The figure 3 we presented is the original research picture.

We are so sorry that I forget to provide new picture in the manuscript.

The data of κ value and program are checked and verified. Based on more κ value from horizontal components of all recordings, we re-fitted the data points linearly and obtained a linear form in research, but we did not provide these.

The new figure 3 we plot are given as the following, and we provide these in the second round revision.

In the new figure 3 ,the kappa value are re-calculated and the line are re-plot. The new linear fitted lines are given.

3. The authors claim that they replaced “improved method” with “improved model”, but I believe that the improved model is also inappropriate. Why does the word “improved” have to be used when the author has not made any improvements to the method or parameter model? Some improvements to the EXSIM method have been made in the following papers, these may be helpful to you.
   (1) Slip-correlated high-frequency scaling factor for stochastic finite-fault modeling of ground motion. Bulletin of the Seismological Society of America, 2022, 112(3):1472-1482.
   (2) Simulation of earthquake ground motion via stochastic finite-fault modeling considering the effect of rupture velocity. Stochastic Environmental Research and Risk Assessment, 2023, 37:2225-2241.
   (3) An updated stochastic finite fault modeling: Application to the Mw 6.0 earthquake in Jiashi, China. Soil Dynamics and Earthquake Engineering, 2022, 162:107450..

**Response:** Thanks for the valuable comment. We have read papers the reviewer suggested in the comments.

We fully agree with the reviewers’ assessment.

According to the reviewers’ advice, the expressions such as “improved method” or “improved model” have been deleted or changed in full paper. On the basis of reading and revising the entire manuscript, the expressions such as “improved method” or “improved model” have been revised.

Please refer to the revised manuscript for details on multiple revisions

4. The most serious problem is the kappa estimation. In the manuscript, equation (6) and equation defined in line 238 were mentioned. Which one was used? In addition, equation defined in line 238 is completely wrong, which should be κ = –k/(πloge) and was used in cartesian coordinate system with data [A, f]. Equation (6) was used in the semi-logarithmic coordinate system with data [lnA, f].

**Response:** Thanks for your valuable and insightful suggestion.

In the kappa estimation, we used the equation (6), rather than equation defined in line 238.

In fact, I can not tell the two formulations apart.

The equation defined in line 238 comes from the literature

(3) An updated stochastic finite fault modeling: Application to the Mw 6.0 earthquake in Jiashi, China. Soil Dynamics and Earthquake Engineering, 2022, 162:107450..

In our paper, the expression of the sentence is referred.

In revised manuscript, the sentence in line 238 has been deleted.

~~In Fourier amplitude spectrum diagram, the~~ $\text{κ}$ ~~of one ground motion recording is computed by~~ $\text{κ}$~~=k/(~~$\pi$~~loge), where k is the slope and e represents natural constant.~~

As mentioned by reviewer, we use Equation (6) in the semi-logarithmic coordinate system with data [lnA, f].
